# Hypoxia and hypotension following industrial-grade anhydrous ethanol ablation of the vein of Marshall in the treatment of atrial fibrillation: a case report

**DOI:** 10.11604/pamj.2024.49.37.44226

**Published:** 2024-10-11

**Authors:** Qijun Zhang, Feiqin Shi, BingJie Song, YingChun Bao, Yong Cao

**Affiliations:** 1 Cardiovascular Department, The Affiliated People´s Hospital of Ningbo University, Ningbo, 315000, ZheJia, China,; 2 Ningbo Yinzhou No.2 Hospital Community, Ningbo, 315000, ZheJia, China

**Keywords:** Atrial fibrillation, vein of Marshall, hypoxia, hypotension, case report

## Abstract

This case presents a patient who experienced hypoxia and hypotension following the infusion of industrial-grade anhydrous ethanol into the vein of Marshall (VOM) during atrial fibrillation radiofrequency ablation. The hypotension lasted for at least three days, requiring dopamine support, while hypoxia persisted for over a week. The prolonged nature of these symptoms posed a diagnostic challenge. A thorough review of the patient's medications and an extensive literature search suggested that the use of industrial-grade anhydrous ethanol may have been the cause. This case highlights the potential risks associated with the use of non-medical grade substances in clinical procedures, emphasizing the importance of careful material selection to avoid severe complications.

## Introduction

The Marshall vein (VOM), due to its embryological specificity and unique anatomical-functional characteristics, has been recognized for its significant involvement in the initiation and perpetuation of atrial fibrillation (AF) and other atrial arrhythmias [1]. While endocardial radiofrequency ablation stands as a crucial therapeutic intervention for AF, its efficacy in eliminating the arrhythmogenic potential mediated by the VOM in certain patients has shown limitations. This limitation provides an anatomical and physiological rationale for the recurrence of AF and the persistence of atrial arrhythmias. Chemical ablation, specifically the administration of anhydrous ethanol into the VOM, emerges as a more definitive technique targeting the atrial musculature associated with the VOM, which can effectively isolate pulmonary vein potentials. This approach creates favorable conditions for subsequent catheter radiofrequency ablation, mitigating postoperative leak points and enhancing the isolation rate of pulmonary vein potentials during AF radiofrequency ablation, thus reducing the incidence of postoperative atrial tachycardia and AF recurrence associated with the VOM. We report a case in which a patient developed hypoxia and hypotension following VOM ethanol infusion for persistent AF, which required dopamine and supplemental oxygen for stabilization before gradual recovery over the subsequent ten days.

## Patient and observation

**Patient information:** a 70-year-old male presented with recurrent palpitations for over a year and bilateral lower limb edema for one week, with a history of atrial fibrillation diagnosed ten days prior. He was treated symptomatically with rivaroxaban and metoprolol, and a routine ECG at our outpatient department showed rapid atrial fibrillation.

**Clinical findings:** during the physical examination, vital signs were recorded as follows: temperature, 36.3°C; pulse, 92 beats per minute; respiration, 18 breaths per minute; blood pressure, 115/67 mmHg; with no jugular venous distention; clear lung fields bilaterally without rales or wheezes; heart rate, 101 beats per minute with absolute irregularity; no pathological murmurs; and no lower limb edema.

**Timeline of current episode:** the patient was referred and admitted to our unit on 24^th^ February 2024. Catheter ablation and anhydrous ethanol infusion VOM were done on 28^th^ February 2024. Hospital discharge on 1^st^ March 2024. Post-discharge follow-up was conducted on the 3rd, 10th, and 45^th^ days after discharge.

**Diagnostic assessment:** the physical examination revealed the following vital signs: On February 18, 2024, BNP was 757pg/ml. A routine ECG performed on February 1, 2024, at our hospital indicated an ectopic rhythm consistent with rapid atrial fibrillation with partial intraventricular conduction delay, low voltage in limb leads, and T-wave changes (II, III, aVF) (V5-V6). Cardiac ultrasound measurements revealed a left atrial diameter (end-systole) of 42 mm, left ventricular diameter (end-diastole) of 48 mm, left ventricular diameter (end-systole) of 26 mm, interventricular septal thickness (end-diastole) of 8 mm, left ventricular posterior wall thickness (end-diastole) of 8 mm, and a left ventricular ejection fraction of 65%. The patient was diagnosed with persistent atrial fibrillation, NYHA functional class II heart failure, hypertension, and liver function abnormalities.

### Therapeutic interventions

**Figure 1:**
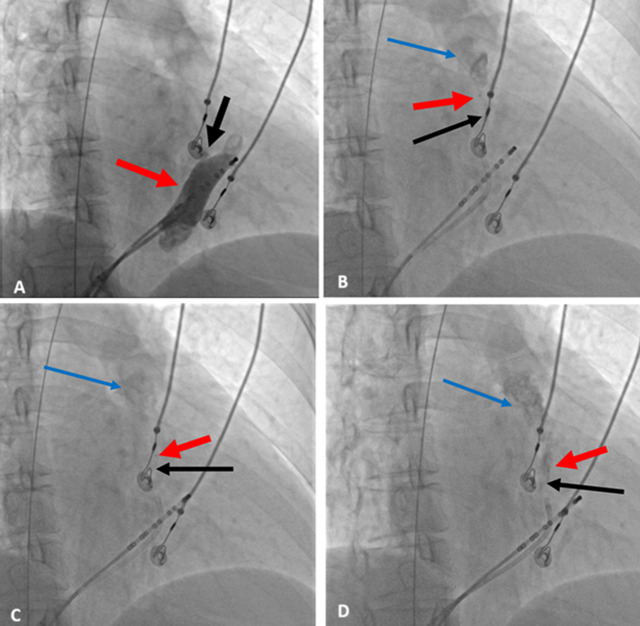
the procedure for anhydrous ethanol ablation as follows; A) illustrates the procedure for coronary sinus venography; B) demonstrates the injection of 2ml of anhydrous ethanol at the distal end of the Vein of Marshall; C) represents the midsection of the vein of Marshall; D) displays the proximal end of the Vein of Marshall; the red arrow indicates the marker of over-the-wire (OTW) balloon, and the black arrow indicates the Vein of Marshall, and the blue arrow indicates the retention of contrast medium within the vein of Marshall

**Figure 2:**
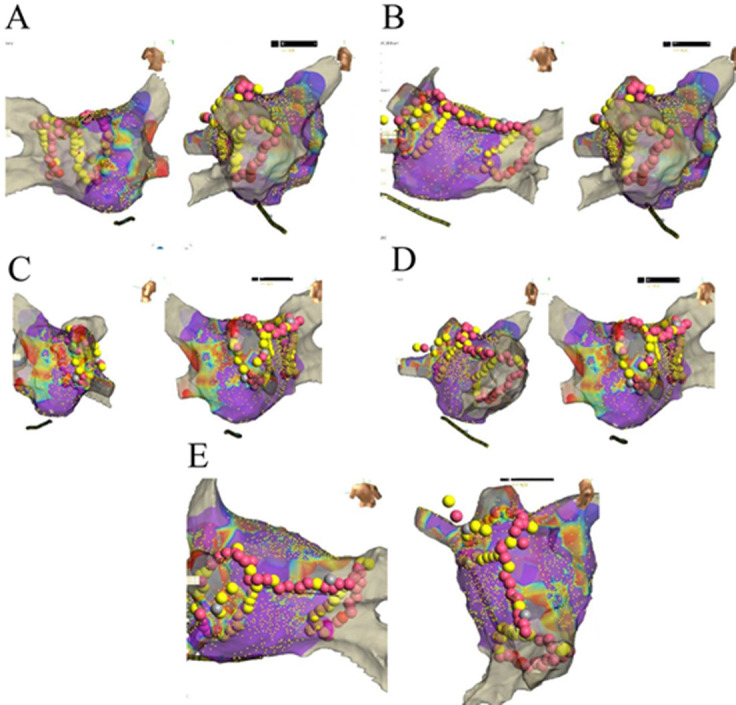
three-dimensional diagram illustrating the successful isolation of pulmonary vein potentials and the ablation of the left atrial roof; A, B) presents the target map for ablation of the right pulmonary vein; C, D) depicts the target map for left circumferential pulmonary vein ablation; E) illustrates the target map for ablation of the left atrial roof line

**Ablation procedure:** after obtaining informed consent, the patient underwent catheter ablation in a supine position with local anesthesia. A guide wire and locking sheath were inserted into the left femoral vein, followed by the successful placement of a controllable electrode into the coronary sinus. A double-lumen balloon catheter was used to deliver 8ml of anhydrous ethanol in increments to the vein of Marshall (VOM), gradually infusing from the distal to proximal segments, with angiography confirming catheter positioning throughout the procedure [2] (Figure 1). Subsequently, pulmonary vein angiography was performed to continue the ablation process. During the procedure, the patient's blood pressure dropped to 82/54mmHg, although no pericardial effusion was detected on echocardiography. Dopamine infusion was initiated to stabilize blood pressure. After stabilization of vital signs, LASSO electrodes and ablation catheters were connected to a three-dimensional mapping system and cold saline perfusion ablation system. Heparin was administered for anticoagulation. Ablation treatment of the left and right pulmonary veins was performed under the guidance of the three-dimensional mapping system at a power setting of 30-35W and a temperature of 43 degrees. Following successful isolation of the pulmonary vein potential, ablation of the atrial top line was pursued (Figure 2). Despite the initial presence of atrial fibrillation rhythm post-ablation, synchronous electrical cardioversion at 200J effectively restored sinus rhythm. Subsequent procedures included a successful blockade of the left atrial posterior wall and mapping of the left atrial subventricular matrix. Although no evident scar tissue was identified on the posterior wall, the fragmented potential was observed near the mitral annulus on the anterior wall. Atrial S1S1 pacing up to 240ms did not induce atrial fibrillation or atrial arrhythmias.

Following surgery, the sheath was promptly removed, and local compression was applied to control bleeding. The patient reported no specific discomfort and vital signs remained stable, with a body temperature of 36.4 °C, a heart rate of 75 beats/min, respiration at 16 breaths/min, and blood pressure at 93/55mmHg. Electrocardiogram monitoring confirmed the presence of sinus rhythm. The surgical procedure proceeded smoothly, and the patient did not report any discomfort. During the post-ablation period, the patient underwent multiple pericardial re-examinations, all of which revealed no evidence of pericardial effusion. However, on the second and third postoperative days, the patient required dopamine to maintain blood pressure due to hypotension. Hypoxic conditions were evident, manifested by cyanosis of the lips and confirmed by low oxygen partial pressure and saturation, as indicated by repeated blood gas analyses and pulse oximetry. Notably, D-dimer levels remained within normal limits, and enhanced chest computed tomography (CT) showed no signs of pulmonary embolism. Despite medical advice to the contrary, the patient opted for discharge on the third postoperative day.

**Follow-up and outcome of interventions:** three days post-discharge: The patient remained asymptomatic with a pulse oximetry reading of 90%, and a blood gas analysis was performed. Ten days post-discharge: the patient remained asymptomatic, exhibiting a blood pressure of 178/78 mmHg, a pulse rate of 70 beats per minute (bpm), a pulse oximetry reading of 99%, a regular heart rhythm with a heart rate of 70 bpm, and no lower limb edema. Forty-five days post-discharge: the patient continued to be asymptomatic, with a blood pressure of 131/73 mmHg, a pulse rate of 81 bpm, a pulse oximetry reading of 99%, a regular heart rhythm with a heart rate of 81 bpm, and no lower limb edema.

**Patient perspective:** the patient was delighted with the quality of care.

**Informed consent:** written informed consent was obtained from the patient for the publication of this case report.

## Discussion

In our case, the patient experienced unexplained persistent hypoxia and hypotension following chemical ablation of the vein of Marshall (VOM) with anhydrous ethanol. The patient had no prior history of pulmonary disease, and preoperative echocardiography revealed no congenital heart disease. D-dimer levels and enhanced chest CT did not support a diagnosis of pulmonary embolism. Despite transient intraoperative hypotension, blood pressure increased with dopamine administration, while hypoxic conditions persisted.

Our investigation into the etiology of sustained hypotension considered the following: we conducted repeated DSA examinations intraoperatively and found no evidence of pericardial effusion. Intraoperative cardiac ultrasound also failed to detect pericardial effusion, and multiple postoperative pericardial re-examinations were unremarkable, leading us to initially exclude hypotension due to surgical complications. We considered the possibility of allergic shock induced by Iohexol contrast agent. However, the patient's blood pressure responded to dopamine, and the use of Iohexol contrast agent in postoperative enhanced chest CT did not result in allergic shock, indicating insufficient evidence to support this hypothesis.

**Figure 3:**
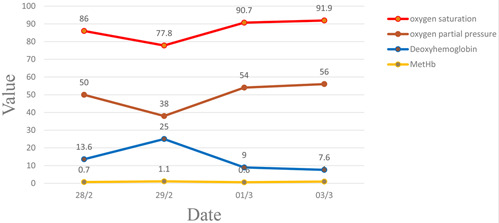
correlation between oxygen partial pressure and oxygen saturation, as well as the concentration of deoxyhemoglobin in the postoperative blood gas analysis of the patient

The prolonged state of hypoxia and hypotension during the postoperative period presented a considerable diagnostic challenge. Particularly noteworthy was the abnormal elevation in deoxyhemoglobin (HHb) levels revealed by blood gas analysis, capturing our attention. We investigated the correlation between oxygen partial pressure and oxygen saturation, as well as the concentration of HHb in the postoperative blood gas analysis of the patient. Surprisingly, we observed a divergent trend in their levels, while Methemoglobin showed no significant alteration (Figure 3). We examined the other main medications administered at the time, excluding contrast agents, which included fentanyl, midazolam, lidocaine, and anhydrous ethanol. Fentanyl typically does not directly affect hemoglobin and is not associated with increased levels of methemoglobin in the blood. Although excessive use of fentanyl or co-administration with other medications can lead to respiratory depression and even respiratory arrest, potentially causing hypoxia, it is not a primary cause of increased methemoglobin levels [3]. Although excessive use of fentanyl or co-administration with other medications can lead to respiratory depression and even respiratory arrest, potentially causing hypoxia, it is not a primary cause of increased methemoglobin levels. Midazolam, a benzodiazepine commonly used for the treatment of seizures or anxiety disorders, does not have a direct relationship with hemoglobin and is unlikely to directly affect methemoglobin levels in the blood [4].

Administration of lidocaine has the potential to induce methemoglobinemia, an abnormal variant of hemoglobin, also known as ferrous hemoglobin, typically formed after exposure to specific chemicals [5,6]. Agents such as nitrites, nitrates, certain medications like amphetamines and phenothiazines, carbon monoxide poisoning, sulfides, various industrial chemicals, pesticides, or organic solvents are known to elevate methemoglobin levels in the blood. In this case, the patient received lidocaine during a transesophageal echocardiogram the day before surgery. However, arterial blood gas analysis showed no significant changes in methemoglobin levels, indicating a low likelihood of persistent hypoxia and hypotension in this instance.

Anhydrous ethanol, while not directly impacting hemoglobin to elevate methemoglobin levels, can exert various effects on the body, some of which may indirectly contribute to increased methemoglobin levels [7]. One of ethanol's metabolic byproducts, acetaldehyde, can bind to hemoglobin and generate methemoglobin [8]. However, this phenomenon typically does not lead to a significant rise in methemoglobin levels in the bloodstream. Overall, although high concentrations of alcohol can induce physiological changes, the likelihood of directly causing a substantial increase in methemoglobin levels in the blood is low.

We considered the possibility of acetaldehyde dehydrogenase deficiency. Acetaldehyde dehydrogenase deficiency is a rare genetic condition that impairs the metabolism of ethanol to acetic acid [9]. Alcohol dehydrogenase (ALDH) enzymes metabolize ethanol to acetaldehyde and then to acetic acid [10]. Patients with acetaldehyde dehydrogenase deficiency may experience hypotension after alcohol consumption. This occurs because the metabolism of ethanol to acetaldehyde causes vasodilation, leading to a decrease in blood pressure. Concurrently, high concentrations of acetaldehyde also increase methemoglobin levels, impacting oxygen carriage and release, resulting in persistent hypoxemia. The diagnosis of this condition involves measuring acetaldehyde concentrations, typically done through blood, urine, or exhaled breath analysis. However, since the patient refrained from alcohol postoperatively, assessing acetaldehyde levels currently isn't feasible. The patient's postoperative blood gas analysis revealed hypoxemia accompanied by elevated levels of deoxyhemoglobin (HHb), rather than methemoglobin, which decreased as oxygenation improved. We conducted a follow-up telephone inquiry with the patient regarding their state during regular alcohol consumption, during which they reported no facial flushing, headache, nausea, vomiting, palpitations, or hypotension. Therefore, this does not support the possibility of acetaldehyde dehydrogenase deficiency in the patient.

It's important to note that, due to cost considerations, we typically use industrial-grade ethanol for our procedures. Normally, trace components in industrial-grade anhydrous ethanol are present in very small amounts. These trace components may include minimal quantities of other alcohol isomers, such as methanol (CH3OH) or propanol (C3H7OH), as well as aldehydes, ketones, esters, and other organic compounds. We can't completely rule out allergies caused by industrial-grade anhydrous ethanol, and the impact of these trace components on the patient's persistent hypoxia and hypotension remains unclear. However, the use of industrial-grade anhydrous ethanol in the ablation of the Vein of Marshall for the treatment of atrial fibrillation may indeed induce hypoxia and hypotension.

## Conclusion

This case illustrates the occurrence of hypoxia and hypotension after ethanol ablation of the Vein of Marshall (VOM) for atrial fibrillation treatment. Drawing from our clinical experience, when persistent hypoxia and hypotension arise during the chemical ablation of the VOM using anhydrous ethanol, and provided that surgical complications and pulmonary embolism have been ruled out, the prognosis remains favorable, warranting a conservative, expectant approach to treatment.
